# Prediction of the need for emergency endoscopic treatment for upper gastrointestinal bleeding and new score model: a retrospective study

**DOI:** 10.1186/s12876-022-02413-8

**Published:** 2022-07-11

**Authors:** Yoshihiro Sasaki, Tomoko Abe, Norio Kawamura, Taisei Keitoku, Isamu Shibata, Shino Ohno, Keiichi Ono, Makoto Makishima

**Affiliations:** 1 Department of Gastroenterology, National Disaster Medical Center, 3256 Midoricho, Tachikawa-shi, Tokyo, 190-0014 Japan; 2 Division of Biochemistry, Department of Biomedical Sciences, Nihon University School of Medicine, 30-1 Oyaguchi-kamicho, Itabashi-ku, Tokyo, 173-8610 Japan

**Keywords:** Bleeding, Blood pressure, Endoscopy, Hemoglobin, Urea

## Abstract

**Background:**

Gastrointestinal bleeding is one of the major gastrointestinal diseases. In this study, our objective was to compare Glasgow-Blatchford score (GBS), AIMS65 score, MAP score, Modified GBS, and Iino score as outcome measures for upper gastrointestinal bleeding. In addition, we extracted factors associated with hemostatic procedures including endoscopy, and proposed a new robust score model.

**Methods:**

From January 2015 to December 2019, 675 patients with symptoms such as hematemesis who visited the National Hospital Organization Disaster Medical Center and underwent urgent upper endoscopy with diagnosis of suspected non-variceal upper gastrointestinal bleeding were retrospectively reviewed. We evaluated the GBS, AIMS65 score, MAP score, Modified GBS, and Iino score, and assessed the outcomes of patients requiring hemostatic treatments at the subsequent emergency endoscopy. We performed logistic regression analysis of factors related to endoscopic hemostasis and upper gastrointestinal bleeding, created a new score model, and evaluated the prediction of hemostatic treatment and mortality in the new score and the existing scores.

**Results:**

The factors associated with endoscopic treatment were hematemesis, heart rate, HB (hemoglobin), blood pressure, blood urea nitrogen (BUN). Based on these predictors and the partial regression coefficients, a new score named H3B2 (using the initial letters of hematemesis, heart rate, HB, blood pressure, and BUN) was generated. H3B2 score was slightly more discriminatory compared to GBS and Modified GBS (area under the receiver operating characteristic curves (AUROC): 0.73 versus 0.721 and 0.7128, respectively) in predicting hemostatic treatment in emergency endoscopy. The H3B2 score also showed satisfactory prediction accuracy for subsequent deaths (AUROC: 0.6857. P < 0.001).

**Conclusions:**

We proposed a new score, the H3B2 score, consisting of simple and objective indices in cases of suspected upper gastrointestinal bleeding. The H3B2 score is useful in identifying high-risk patients with suspected upper gastrointestinal bleeding who require urgent hemostatic treatment including emergency endoscopy.

## Background

Gastrointestinal bleeding is one of the major gastrointestinal diseases. Upper gastrointestinal bleeding accounts for more than 50% of all gastrointestinal bleeding and related hospitalizations [1], with a mortality rate estimated to be 2–10% [2, 3]. The most common cause of acute upper gastrointestinal bleeding is non-variceal upper gastrointestinal bleeding.

Urgent endoscopy is performed for suspected gastrointestinal bleeding at night or on holidays, but many cases are experienced in which the bleeding has already stopped spontaneously at the time of endoscopy. Various guidelines indicate the need to stratify patients with gastrointestinal bleeding into high-risk cases requiring immediate treatment before endoscopy and low-risk cases that do not, along with their management before, during, and after endoscopy [4, 5]. If the stratification can reduce the number of urgent endoscopic examinations, it will lead to more efficient medical care and less burden on physicians.

The Glasgow-Blatchford score (hereinafter referred to as “GBS”) was reported as an outcome measure for upper gastrointestinal bleeding, which includes comprehensive clinical treatment including blood transfusion, rebleeding, and death in addition to endoscopic treatment [6] (Table 1), and the AIMS65 score, which measures the risk of death in patients with upper gastrointestinal bleeding, was reported [7] (Table 2). Although the GBS and AIMS65 score do not assess the need for endoscopic treatment, they are used to evaluate therapeutic interventions for bleeding because a useful score to predict endoscopic hemostasis is missing.

**Table 1:** GBS

Item	Standard	Score
BUN (mmol/L) (Value in mg/dl)	< 6.5 (< 18.2)	0
	6.5–7.9 (18.2–22.4)	2
	8.0–9.9 (22.5–28.0)	3
	10.0–24.9 (28.1–69.0)	4
	≧25.0 (≧70)	6
HB, male (g/dl)	12–12.9	1
	10–11.9	3
	< 10	6
HB, female (g/dl)	10–11.9	1
	< 10	6
Systolic blood pressure (mmHg)	100–109	1
	90–99	2
	< 90	3
Other indicators	Pulse ≧ 100	1
	Bloody stool	1
	Fainting	2
	Liver disease	2
	Heart disease	2

The need for treatment for upper gastrointestinal bleeding is stratified using BUN, HB, blood pressure, and other indicators, and evaluated on a scale of 0–23 [6]

**Table 2:** AIMS65 score

Item	Standard	Score
Albumin (mg/dl)	< 3.0	1
PT-INR	> 1.5	1
Disturbance of consciousness		1
Systolic blood pressure (mmHg)	< 90	1
Age (years)	> 65	1

In order to easily predict the prognosis of upper gastrointestinal bleeding, 5 indicators of albumin, PT-INR, impaired consciousness, blood pressure, and age are used to evaluate on a score of 0–5 [7]

The GBS has been reported to be useful in the evaluation of therapeutic intervention for bleeding [8–10]. GBSs range from 0 to 23 points [6]. It has been reported that a score of 7 points or higher is useful as an index for endoscopic treatment [8]. A score of 1 point or lower is low risk and does not require intervention such as endoscopic treatment [10, 11]. The higher the score, the more therapeutic intervention is required, but the evaluation of the point at which therapeutic intervention is required has not been established. Redondo-Cerezo et al. proposed the MAP score, which consists six indices: Glasgow Coma Scale score (< 15), American Society of Anesthesiologists score (> 2), pulse (> 100 beats/min), albumin (< 2.5 mg/dl), systolic blood pressure (< 90 mmHg), and hemoglobin (HB) (< 10 g/dl), and reported that it is highly predictive of therapeutic intervention including endoscopy and mortality in upper gastrointestinal bleeding [12] (Table 3). In order to predict the need for therapeutic intervention including endoscopy in upper gastrointestinal bleeding, the Modified GBS consisting of pulse rate, systolic blood pressure, blood urea nitrogen (BUN), and HB is useful [13, 14] (Table 4).

**Table 3:** MAP score

Item	Standard	Score
Disturbance of consciousness	Glasgow Coma Scale < 15	1
American society of anesthesiologists score	> 2	1
Pulse (times/minute)	> 100	1
Albumin (mg/dl)	< 2.5	2
Systolic blood pressure (mmHg)	< 90	2
HB (g/dl)	< 10	2

In order to predict treatment intervention including endoscopy and mortality in upper gastrointestinal bleeding, evaluation is performed on a score of 0–9 using the above items [12]

**Table 4:** Modified GBS

Item	Standard	Score
BUN (mmol/L) (Value in mg/dl)	< 6.5 (< 18.2)	0
	6.5–7.9 (18.2–22.4)	2
	8.0–9.9 (22.5–28.0)	3
	10.0–24.9 (28.1–69.0)	4
	≧25.0 (≧70)	6
HB, male (g/dl)	12–12.9	1
	10–11.9	3
	< 10	6
HB, female (g/dl)	10–11.9	1
	< 10	6
Systolic blood pressure (mmHg)	100–109	1
	90–99	2
	< 90	3
Pulse (times/minute)	≧100	1

In order to predict the clinical outcome of upper gastrointestinal bleeding, only indicators of BUN, HB, systolic blood pressure, and pulse from the GBS are used to evaluate on a score 0–16 [13]

Iino et al. analysed 212 Japanese patients to predict the need for endoscopic treatment excluding blood transfusion. They evaluated the scores and factors that are included in the GBS and AIMS65 score and are likely associated with upper gastrointestinal bleeding, including prothrombin time-international normalized ratio (PT-INR), liver disease, heart failure, renal failure, collagen disease, *Helicobacter pylori* infection, malignancy, antiplatelet agents, anticoagulants, proton pump inhibitors (PPIs) or histamine receptor 2 blockers, corticosteroids, and nonsteroidal anti-inflammatory drugs. Multivariate analysis showed that systolic blood pressure, syncope, hematemesis, HB, BUN, estimated glomerular filtration rate (eGFR), and antiplatelet agents are predictors of the need for therapeutic intervention in gastrointestinal bleeding, and a new scoring model was created (hereafter referred to as the Iino score) [15] (Table 5). However, the Iino score has not been widely used in clinical practice due to the lack of validation and the difficulty in interviewing all patients about syncope and antiplatelet medication before endoscopy.

**Table 5:** Iino score

Item	Standard	Score
Systolic blood pressure (mmHg)	< 100	2
Syncope	2	2
Hematemesis		3
HB (g/dl)	< 10	1
BUN (mmol/L)	≧22.4	2
eGFR (mL/ min/1.73m^2^)	≧60	− 2
Oral antiplatelet drug		− 2

In order to predict the indication of endoscopic treatment for upper gastrointestinal bleeding, the above items are used to evaluate on a score of − 4 to 10 [15]

We evaluated the usefulness of a scoring system for the risk of gastrointestinal bleeding using the GBS, AIMS65 score, MAP score, Modified GBS, and Iino score. The usefulness of the scoring system for gastrointestinal bleeding risk was evaluated. We extracted factors related to endoscopic treatment and created a new score that is simpler and more useful. In addition, we compared the usefulness of the new score and the existing prediction score in predicting hemostatic therapy in patients with non-variceal upper gastrointestinal bleeding and the accuracy of predicting mortality.

## Methods

### Patient management

**Fig. 1:**
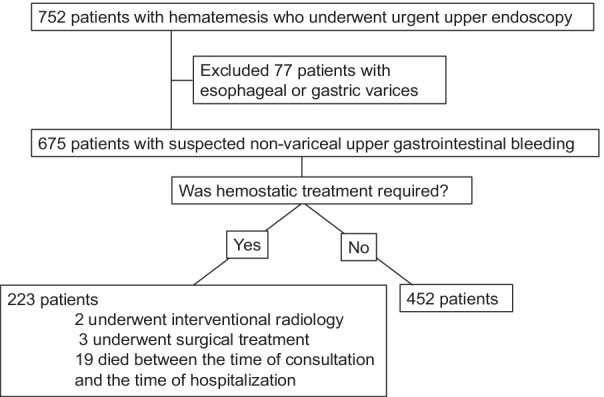
Patient flow diagram

From January 2015 to December 2019, 752 patients with symptoms of upper gastrointestinal bleeding, such as hematemesis, black stool, fainting, and anemia, who were transported to the National Disaster Medical Center of the National Hospital Organization or visited the outpatient clinic, and underwent upper gastrointestinal endoscopy within 6 h of the visit, with upper gastrointestinal bleeding suspected based on symptoms and blood tests, were retrospectively reviewed. A total of 675 patients with suspected non-variceal upper gastrointestinal bleeding were included in the study, excluding patients with esophageal or gastric varices (Fig. 1). Blood samples were taken immediately after the visit, and clinical symptoms were interviewed at the time of the visit or before treatment. The GBS, AIMS65 score, MAP score, Modified GBS, and Iino score were calculated to determine whether endoscopic hemostatic treatment or other hemostatic treatment was necessary at the subsequent emergency endoscopy and whether death occurred afterwards. Endoscopic hemostasis, such as clipping, hypertonic saline-epinephrine injection, argon plasma coagulation, and absolute ethanol injection, was performed according to the guidelines of the Japanese Society of Gastrointestinal Endoscopy [11]. The hemostatic treatment of ulcerative lesions was performed according to Forrest's modified classification of active bleeding (Ia: eruptive bleeding, Ib: exudative bleeding) and IIa (with guttural bleeding vessels) among recent bleeding [16]. Patients who underwent endoscopic hemostasis were diagnosed with gastric/duodenal ulcer, Mallory-Weiss syndrome, hemorrhagic gastritis, or malignant tumor.

This retrospective study was approved by the Hospital Ethical Review Committee at the National Disaster Medical Center in December 2020 (National Disaster Medical Center: number 2020–17) and by the Clinical Research Ethics Review Committee at Nihon University Medical College Itabashi Hospital in February 2021 (Nihon University Medical College Itabashi Hospital: number RK-210209–9). The treatments performed were part of the current standard of care, and patient data were anonymized. Informed consent was exempted by Clinical Research Ethics Review Committee at Nihon University Medical College Itabashi Hospital due to the retrospective nature of the study. All methods were performed in accordance with the relevant guidelines and regulations.

### Data collection

Patient data were collected by the authors and a full-time research clerk. The collected data included patient characteristics, clinical symptoms, blood tests, and treatment actions necessary to calculate each score. Endoscopic treatment, interventional radiology, surgery, and death were recorded (Table 6).

**Table 6:** Baseline data of patients undergoing emergency endoscopy in 2015–2019

Item	Numerical value
Mean age (years)	72.3 ± 14.5
Men/women	414 (61%)/261 (39%)
Systolic/diastolic BP	123.3 ± 23.7/68.2 ± 13.4
Pulse	84.1 ± 18.8
History (heart/liver disease)	164 (24%)/43 (6%)
Antiplatelets/anticoagulants	133 (20%)/124 (18%)
HB	9.1 ± 2.7
BUN	36.4 ± 28.5
Alb	2.8 ± 1.4
eGFR	59.7 ± 31.7
INR	1.0 ± 0.8
Impaired consciousness	47 (7.0%)
Melena	383 (56.7%)
Fainting	28 (4.1%)
Hematemesis	167 (24.7%)
*Helicobacter pylori* positive	107 (15.9%)
Nonsteroidal anti-inflammatory drugs takers	61 (9.0%)
Steroid users	16 (2.4%)
PPI oral	187 (27.7%)
*Helicobacter pylor*i eradication treatment history	6 (0.9%)
Endoscopic hemostasis therapy (including unsuccessful)	223 (33.0%)
Interventional Radiology	2 (0.3%)
Surgical treatment	3 (0.4%)
Death	19( 2.8%)

### Statistical analysis

Optimal score thresholds for predicting low-risk patients who may not require hemostatic treatment were identified based on a sensitivity of > 95%. The relationship between each item and endoscopic treatment was evaluated using binomial logistic analysis to determine which of the items was associated with hemostatic treatment. Significantly related factors were extracted, and a new score associated with hemostasis (H3B2 score named using the initial letters of hematemesis, heart rate, HB, blood pressure, and BUN; explained in the Result section) was created by considering partial regression coefficients. Next, we stratified the number of patients who received hemostatic treatment or not according to the five existing gastrointestinal bleeding risk score systems (GBS, AIMS65 score, MAP score, Modified GBS, and Iino score) and the new score (H3B2 score) according to each risk score system.

We also used area under the receiver operating characteristic curves (AUROC) to compare the ability of each of the existing scores to predict the need for endoscopic treatment with the new score. We validated the cutoff value of the new score and compared the ability of each existing score and the new score to predict subsequent death using AUROC.

## Results

### Patient characteristics and baseline scores

A total of 675 patients were included in this study (Fig. 1). The mean age was 72.3 years, and the male to female ratio was about 6:4. 24% had a history of cardiac disease, and 6% had a history of liver disease. 57% of the patients showed hemorrhage and black stools, 25% showed hematemesis, and 7% showed disturbance of consciousness. All 223 patients who underwent endoscopy required hemostatic treatment. 2 underwent interventional radiology in addition to endoscopic treatment and 3 underwent surgical treatment. 19 patients died between the time of consultation and the time of hospitalization (Table 6).

### Factors associated with gastrointestinal bleeding

The GBS consists of BUN, HB, systolic blood pressure, pulse rate, hypovolemia/black stools, syncope, cardiac and cardiovascular disease [7]. The AIMS65 score consists of albumin, PT-INR, mental and consciousness disorders, systolic blood pressure, and age [8]. The Iino score consists of items such as systolic blood pressure, syncope, hematemesis, HB, BUN, eGFR, and antiplatelet agents [10]. We conducted a logistic analysis to evaluate whether the individual factors comprising the above three risk scoring systems were related to endoscopic hemostatic therapy in patients with gastrointestinal bleeding. Factors associated with hemostatic treatment were blood pressure (P = 0.0283), BUN (P < 0.001), HB (P = 0.0037), hematemesis (P = 0.0030), and pulse (P = 0.0137) were the factors directly related to hemostatic treatment in emergency endoscopy (9) (Table 7). We generated a new score named H3B2 (using the initial letters of hematemesis, heart rate, HB, blood pressure, and BUN) by determining score component values (1 for hematemesis, heart rate, blood pressure and HB; 2 for BUN) (Table 8) based on the partial regression coefficients (hematemesis, 0.6682; heart rate (pulse), 0.5729; blood pressure, 0.6954; HB, 0.5902; BUN, 1.0484 (1.6 to 1.8-fold compared to other factors) (Table 7).

**Table 7:** Binary logistic regression model to determine the independent association of factors involved in gastrointestinal bleeding on the undertaking hemostatic treatment upon endoscopy

Variable	Partial regression coefficient	Standard error	Standardized partial regression coefficient	Partial regression coefficient 95% confidence interval			Odds ratio 95% confidence interval		Partial regression coefficient significance test			*: P < 0.05 **: P < 0.01
				Lower endpoint	Upper endpoint	Odds ratio	Lower endpoint	Upper endpoint	Wald	Degree of freedom	P-value	
Liver disease	− 0.3078	0.3954	− 0.0752	− 1.0828	0.4673	0.7351	0.3386	1.5956	0.6058	1	0.4364	
Heart failure	− 0.1034	0.2442	− 0.0444	− 0.5821	0.3752	0.9017	0.5587	1.4553	0.1794	1	0.6719	
Antiplatelets	0.0382	0.2322	0.0152	− 0.4170	0.4934	1.0389	0.6590	1.6379	0.0270	1	0.8694	
Anticoagulants	0.1142	0.2555	0.0442	− 0.3866	0.6150	1.1209	0.6793	1.8496	0.1996	1	0.6550	
Mental illness, impaired consciousness	0.1122	0.3422	0.0285	− 0.5586	0.7829	1.1187	0.5720	2.1878	0.1074	1	0.7431	
Melena	0.3794	0.1947	0.1880	− 0.0022	0.7610	1.4614	0.9978	2.1403	3.7970	1	0.0513	
Fainting	0.7034	0.4236	0.1402	− 0.1268	1.5335	2.0205	0.8809	4.6346	2.7573	1	0.0968	
Hematemesis	0.6682	0.2254	0.2883	0.2265	1.1098	1.9506	1.2542	3.0339	8.7908	1	0.0030	**
Pulse	0.5729	0.2324	0.2124	0.1173	1.0285	1.7734	1.1245	2.7968	6.0746	1	0.0137	*
Albumin (≤ 3.0)	− 0.0054	0.1892	− 0.0026	− 0.3762	0.3654	0.9946	0.6865	1.4411	0.0008	1	0.9773	
INR (≥ 1.5)	− 0.0233	0.1948	− 0.0110	− 0.4051	0.3584	0.9769	0.6669	1.4310	0.0144	1	0.9046	
Blood pressure (≤ 90)	0.6954	0.4924	0.1478	− 0.2698	1.6605	2.0044	0.7635	5.2620	1.9941	1	0.1579	
Age (≥ 65 years)	0.0089	0.2279	0.0039	− 0.4377	0.4555	1.0090	0.6455	1.5770	0.0015	1	0.9688	
Blood pressure (≤ 100)	0.6599	0.3008	0.2233	0.0703	1.2495	1.9345	1.0728	3.4885	4.8116	1	0.0283	*
HB (≤ 10.0)	0.5902	0.2035	0.2848	0.1914	0.9890	1.8043	1.2110	2.6885	8.4148	1	0.0037	**
BUN (≥ 22.4)	1.0484	0.2057	0.5133	0.6453	1.4515	2.8530	1.9065	4.2694	25.9843	1	0.001	**
eGFR (≥ 60)	0.0091	0.2003	0.0045	− 0.3835	0.4017	1.0091	0.6815	1.4944	0.0021	1	0.9637

**Table 8:** H3B2 score, a new score for prediction of the indication of hemostasis treatment

Item	Standard	Score
Hematemesis		1
Heart rate (times/minute)	≧100	1
Blood pressure (mmHg)	≦100	1
HB (g/dl)	≦10	1
BUN (mg/dl)	≧22.4	2

In order to predict the indication of hemostasis treatment including endoscopic treatment in upper gastrointestinal bleeding, the above items are used to evaluate on a score of 0–6

### Comparison of the existing score and the new score in terms of their ability to predict the need for hemostatic treatment and subsequent death during emergency endoscopy

**Fig. 2:**
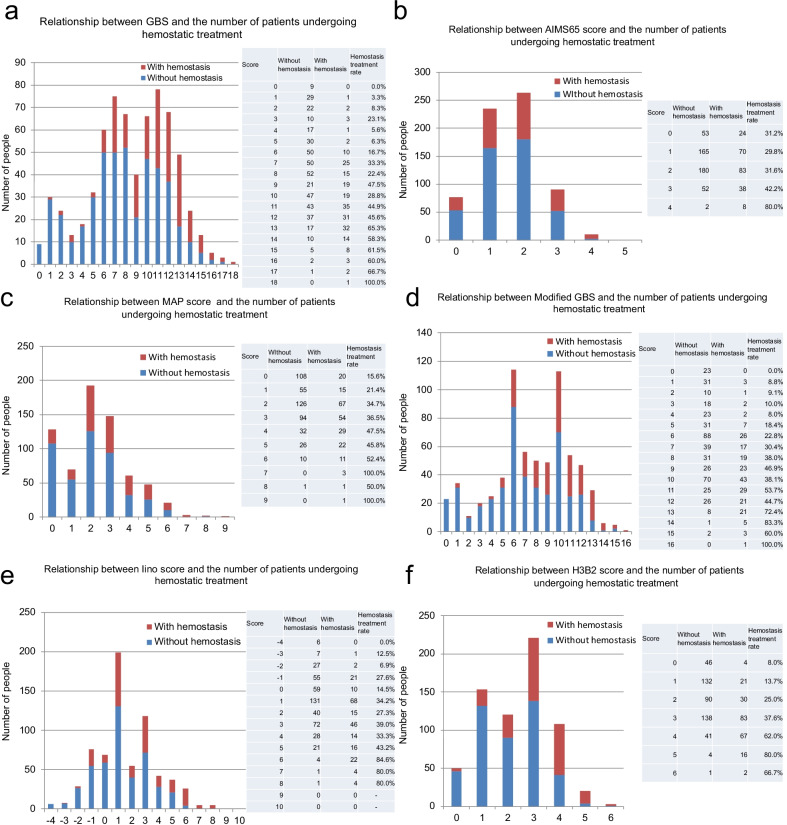
Number of patients with and without hemostasis treatment by scoring systems of the GBS (**a**), AIM65 score (**b**), MAP score (**c**), Modified GBS (**d**), Iino score (**e**) and H3B2 score (**f**)

**Fig. 3:**
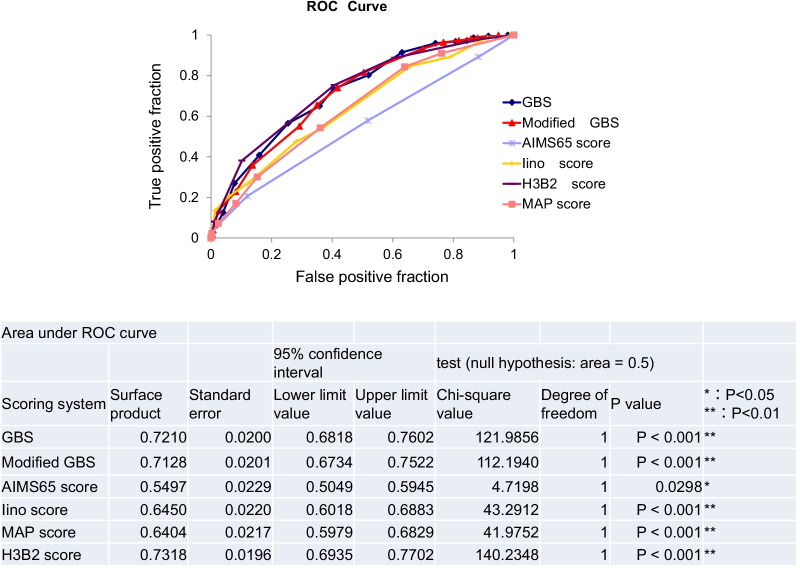
ROC curves comparing the prediction of hemostatic treatment on the GBS, Modified GBS, AIMS65 score, Iino score, MAP score, and H3B2 score

**Fig. 4:**
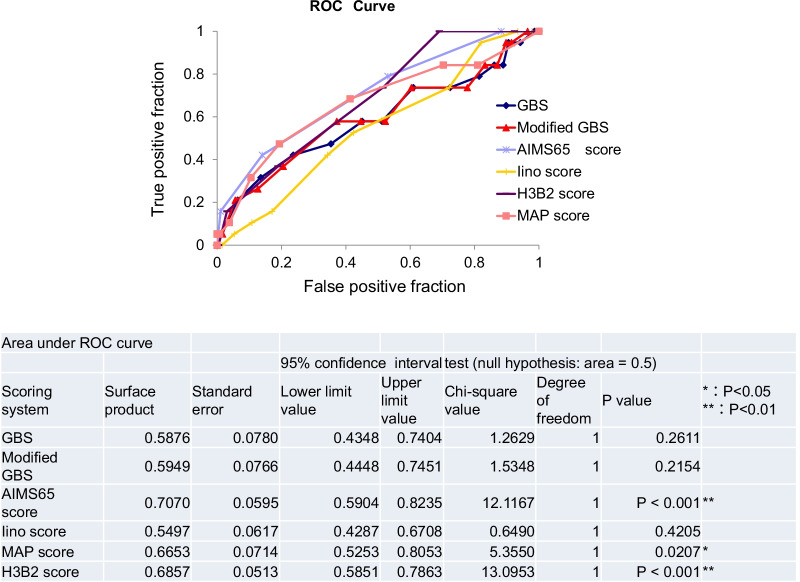
ROC curves comparing the prediction of mortality on the GBS, Modified GBS, AIMS65 score, Iino score, MAP score, and H3B2 score

Figure 2 represents the relationship between the number of patients undergoing hemostatic treatment and each of the 5 score analysed (Fig. 2). The receiver operating characteristic (ROC) curve was used to evaluate the usefulness of the existing score and the new H3B2 score (Table 9). In the existing model, GBS and Modified GBS showed higher discriminatory ability (AUROC: 0.721 and 0.7128, respectively; P < 0.001) in predicting hemostatic treatment in emergency endoscopy compared with other existing scores, and the new H3B2 score was even more discriminatory (AUROC: 0.73). The optimal cutoff value for the H3B2 score was 3 points (Fig. 3). The H3B2 score also showed higher prediction accuracy for subsequent deaths (AUROC: 0.6857. P < 0.001), although it was inferior to AIMS65 (AUROC: 0.7070; P < 0.001); P < 0.001) (Fig. 4).

**Table 9:** ROC curve data (score associated with hemostasis)

Reference point	True positive fraction	False positive fraction	Odds ratio	Specificity
0	1.0000	1.0000	–	0
1	0.9821	0.8982	6.2032	0.1018
2	0.8879	0.6062	5.1451	0.3938
3	0.7534	0.4071	4.4490	0.5929
4	0.3812	0.1018	5.4364	0.8982
5	0.0807	0.0111	7.8498	0.9889
6	0.0090	0.0022	4.0814	0.9978

The optimal cutoff value for the H3B2 score was 3 points

## Discussion

Hemostasis during emergency endoscopy has become easier than before with the advancement of instruments, and a high success rate has been maintained [17, 18]. However, spontaneous hemostasis may occur during emergency endoscopy, and endoscopic hemostasis is not necessary in all cases [19].

In cases of suspected acute gastrointestinal bleeding, early risk stratification to identify high-risk patients is important and is associated with the timing and need for subsequent endoscopy. In an international multicenter prospective study of more than 3000 patients, GBS was the best predictor of the need for hospital-based intervention or death [8]. A GBS of 7 or higher indicated the need for endoscopic intervention. Another report showed that a GBS of 12 points or higher had a 90% specificity for predicting in-hospital mortality, and that a delay in endoscopy significantly increased the number of deaths in patients with a GBS of 12 points or higher [20]. The GBS was useful for endoscopic intervention [21] and hemostatic treatment [22] among conventional scores. The GBS correlates with therapeutic intervention and lethality as the score increases, but the actual score at which therapeutic intervention should be performed has not been determined. A systematic review of 16 reports confirmed that the GBS was better than other cutoff points and risk scores at identifying low-risk patients, but had very low specificity [10]. Therefore, it is difficult to accurately assess the risk of patients with upper gastrointestinal bleeding to determine what cases should be promptly endoscoped and treated [23].

Therefore, we conducted this study to examine the association between each existing score and the need for endoscopic or other hemostatic treatment. The GBS was found to be the most useful in predicting hemostatic treatment, as previously reported. However, there are many items in the GBS, and it is difficult to assess accurately in emergency medicine because it includes subjective factors such as fainting and the presence of cardiac and hepatic diseases. In addition, it is difficult to define cardiac and hepatic diseases, and it is a difficult question whether to include even minor diseases.

From our study, the factors associated with endoscopic treatment were hematemesis, heart rate (> 100 beats/min), HB (10.0 g/dl or less), blood pressure (100 mmHg or less), BUN (22.4 mg/dl or higher). Hematemesis can be observed from the surroundings and can be easily and objectively assessed by examining the oral cavity and perioral area. Therefore, the above five factors are all objective indicators. The measurement of each clinical index is simple and can be performed in actual clinical situations requiring emergency. Based on the above predictors and the partial regression coefficients, we proposed a new score with a total of 6 points, 2 points for BUN and 1 point for each of the other factors, and named this score the H3B2 score, using the initial letters of each factor (hematemesis, heart rate, HB, blood pressure, and BUN). The H3B2 score can be used as a useful index compared to GBS and Modified GBS. The H3B2 score has a cutoff value of 3 points, which is the maximum value based on the Youden index, as a guideline for hemostatic treatment. However, considering the clinical aspects, a score of 2 or higher, which has a certain degree of sensitivity, should be considered as an indication for urgent endoscopy and should be a subject for further study. Although the H3B2 score is inferior to the AIMS65, it is also an excellent predictor of subsequent death.

In this study, we did not uniformly administer PPIs between the time of our consultation and the endoscopic intervention, although patients were taking PPIs regularly. North American and European guidelines suggest the use of high-dose PPIs before endoscopy as basic therapy to reduce the incidence of peptic ulcer [24–26]. Patients receiving PPIs prior to endoscopic intervention were significantly less likely to develop adverse outcomes and had significantly lower rates of rebleeding, upper gastrointestinal surgery, mortality, and length of hospital stay compared with patients who did not receive PPIs [27–29]. Potassium-competitive acid blockers (P-CABs) were approved in Japan in 2015, the first in the world. P-CABs do not require acid activation, are stable in acidic environments, and accumulate in secretory tubules in high density. This may be an issue for further study.

Shung et al. validated a machine-learning model for hemostatic treatment requiring hospitalization and 30-day mortality in patients with upper gastrointestinal bleeding using a 24-item index that included demographics, comorbidities, medications, clinical characteristics, and blood sampling results. It showed higher area under ROC curve, sensitivity, and specificity than the conventional GBS and AIMS65 score [30]. However, it requires many items and a detailed interview, and patients themselves often do not remember their medications and comorbidities accurately, so it is not suitable for urgent care at present. However, with the development of AI and digitalization, for more efficient collection of medical information, the Shung et al. score with higher sensitivity and specificity using more indicators may become necessary in the future.

This study has some limitations. First, it was an analysis of cases from a single institution. Further studies are needed for validation of the H3B2 score in external cohorts. Second, patients with esophageal varices were not analysed. Our conclusions cannot be applied to all patients with suspected upper gastrointestinal bleeding. However, there are few data on the use of scoring systems in patients with variceal bleeding, and the predictive power is low. Third, the primary endpoint in this study was the presence or absence of hemostatic treatment in patients who underwent emergency endoscopy within 6 h of presentation. A prospective observational study in Korea evaluated mortality and rebleeding rate 28 days after hospitalization in adult patients with GBS 7 or higher non-variceal upper gastrointestinal bleeding who underwent endoscopy within 6 h of emergency department visit and those who underwent endoscopy within 6–48 h. The mortality rate was significantly lower in the group that underwent endoscopy within 6 h, but there was no difference in rebleeding between the two groups [31]. Another report examined whether emergency upper gastrointestinal endoscopy within 6 h improved all-cause mortality at 30 days compared with early endoscopy within 6 to 24 h in patients with stable upper gastrointestinal hemorrhage without persistent hemorrhage (GBS 12 points or higher). However, it was reported that there was no significant difference in all-cause mortality between the two groups [19]. There is controversy about when endoscopy should be performed in patients with suspected acute upper gastrointestinal bleeding [32]. Even if the new score can predict hemostatic therapy such as endoscopic treatment and mortality, it remains to be determined when therapeutic intervention can improve important clinical outcomes such as death.

## Conclusions

In summary, we proposed a new score, the H3B2 score, consisting of simple and objective indices in cases of suspected upper gastrointestinal bleeding. The H3B2 score is useful in identifying high-risk patients with suspected upper gastrointestinal bleeding who require urgent hemostatic treatment including emergency endoscopy.

## Data Availability

The datasets used and/or analysed during the current study are available from the corresponding author on reasonable request.

## Abbreviations

AUROC: Area under the receiver operating characteristic curves
BUN: Blood urea nitrogen
eGFR: Estimated glomerular filtration rate
GBS: Glasgow-Blatchford score
HB: Hemoglobin
P-CABs: Potassium-competitive acid blockers
PPI: Proton pump inhibitor
PT-INR: Prothrombin time-international normalized ratio
ROC: Receiver operating characteristic
